# Blood serum proteins as biomarkers for prediction of survival, locoregional control and distant metastasis rate in radiotherapy and radio-chemotherapy for non-small cell lung cancer

**DOI:** 10.1186/s12885-019-5617-1

**Published:** 2019-05-08

**Authors:** Rafał Suwinski, Monika Giglok, Katarzyna Galwas-Kliber, Adam Idasiak, Bozena Jochymek, Regina Deja, Barbara Maslyk, Jolanta Mrochem-Kwarciak, Dorota Butkiewicz

**Affiliations:** 1 Radiotherapy and Chemotherapy Clinic and Teaching Hospital, Center of Oncology, M. Sklodowska-Curie Memorial Cancer Center and Institute of Oncology, Gliwice Branch, Wybrzeze Armii Krajowej 15, 44-100 Gliwice, Poland; 2 Department of Radiation Oncology, M. Sklodowska-Curie Memorial Cancer Center and Institute of Oncology, Gliwice Branch, Wybrzeze Armii Krajowej 15, 44-100 Gliwice, Poland; 3 Department of Analytical and Clinical Biochemistry, M. Sklodowska-Curie Memorial Cancer Center and Institute of Oncology, Gliwice Branch, Wybrzeze Armii Krajowej 15, 44-100 Gliwice, Poland; 4 Center for Translational Research and Molecular Biology of Cancer, Center of Oncology, M. Sklodowska-Curie Memorial Cancer Center and Institute of Oncology, Gliwice Branch, Wybrzeze Armii Krajowej 15, 44-100 Gliwice, Poland

**Keywords:** Lung cancer, Radiotherapy, Biomarkers, Survival, Osteopontin, Vascular endothelial growth factor, Erythropoetin, High mobility group box 1 protein, Insulin-like growth factor 1, Platelet-derived growth factor

## Abstract

**Background:**

Several studies have documented that blood biomarkers can improve basic prognostic models in radiotherapy and radio-chemotherapy for non-small cell lung cancer. The current study evaluated the prognostic impact of six markers focusing on their utility in homogenous subsets, compared to the significance in a large heterogeneous group.

**Methods:**

Blood samples of 337 patients who were referred for curative or palliative external beam thoracic radiotherapy for non-small cell lung cancer were collected. The concentration of osteopontin (OPN), vascular endothelial growth factor (VEGF), erythropoetin (EPO), high mobility group box 1 protein (HMGB1), insulin-like growth factor 1 (IGF-1) and platelet-derived growth factor (PDGF) in serum were measured by ELISA assay and the prognostic potential was assessed using univariable and multivariable survival models.

**Results:**

Multivariable analysis revealed that out of several variables studied six dichotomized features: namely: cigarette smoking, lack of chemotherapy, palliative doses of radiotherapy, high OPN concentration, advanced T stage and high VEGF concentration had a highly significant (*p* < 0.005) and independent influence on overall survival in the group of 337 patients. In a subset of patients treated with curative radio-chemotherapy or radiotherapy (*N* = 148) tumor pathology, EPO concentration and VEGF concentration, significantly and independently influenced overall survival. In a subset of patients with squamous cell cancer (*N* = 206) OPN had a highly significant impact on overall survival. In contrast, in a subset of patients with nonsquamous histology (*N* = 131) only VEGF had a significant influence on survival.

**Conclusions:**

Blood serum proteins appear to be clinically useful prognosticators of overall survival in radio-chemotherapy and radiotherapy for non-small cell lung cancer. In unselected heterogeneous groups, dichotomized concentrations of OPN and VEGF emerged among the strongest independent prognosticators of overall survival. VEGF and EPO concentration (dichotomized) were found to be independent prognostic factors among the patients treated with curative doses of radiotherapy. The utility of OPN as a prognostic marker appeared restricted to the patients with squamous histology.

## Background

External beam radiotherapy or radio-chemotherapy is commonly employed in both curative and palliative treatment for non-small cell lung cancer. Several factors influence the outcome of these patients, including TNM stage, age, gender, histologic type of the tumor, radiation dose and the overall radiation treatment time. Precise assessment of the prognosis is essential for adequate selection of treatment strategy and for proper stratification of individuals within prospective trials. Numerous attempts have been made to improve well-established prognostic models that are based on the assessment of clinicopathological features. These attempts include the analysis of circulating tumor cells [1], circulating cell-free tumor DNA [2], gene expression profiles from bronchoscopy obtained tumor samples [3], the analysis of common somatic mutations and rearrangements in specific genes including EGFR, ALK, ROS1, Her2, BRAF, KRAS, PTEN [4] and the analysis of blood serum proteins [5]. The literature on this subject is extensive hence we herein refer to review studies on the relevant topic [1, 2, 4, 5]. We also note that, apart from prognostic utility, blood biomarkers are heavily tested as potential diagnostic markers in the early detection of lung cancer [6, 7].

Most of the studies note that, considering advanced inoperable cases, the amount of tumor tissue in bronchoscopy obtained tumor samples is scarce and, in most circumstances, cautiously stored for assessment of the molecular and pathological biomarkers that are necessary for proper selection of the modern systemic treatment (PDL-1 expression, EGFR and ROS mutations, ALK rearrangement etc..). This finding provides the basic rationale for the analysis of blood biomarkers, because the blood samples can be readily obtained both before, during and after radiotherapy and this process does not interfere with assessment of the tumor biomarkers that are now heavily utilized in medical oncology.

While the literature on the diverse utility of the biomarkers in lung cancer is broad, relatively few studies have directly focused on assign of the independent prognostic value of blood serum proteins as biomarkers of the long-term prognosis in radiotherapy and radio-chemotherapy for non-small cell lung cancer. Nonetheless, the existing studies document that such an approach is promising and may considerably improve basic clinically-oriented prognostic models [8–10]. However, the practical significance and utility of blood serum markers may largely depend on the clinical and therapeutic characteristics of the group of patients. Therefore, we analyzed the selected blood markers in a large group of patients with non-small cell lung cancer treated with radiotherapy or radio-chemotherapy focusing on the utility of these markers in the homogenous subsets, compared to significance in a large heterogeneous group.

## Methods

### General characteristics of the patients

Between September 2006 and May 2015 blood samples from 337 patients who were referred for curative or palliative external beam thoracic radiotherapy for non-small cell lung cancer were collected. Patients who had surgery for lung cancer were excluded. Likewise, patients who did not have thoracic radiotherapy were excluded. The group was heterogeneous with respect to the stage of disease, tumor pathology and general performance status (Table 1).

**Table 1:** Baseline characteristics of 337 patients (including prognostic significance of the selected variables)

Variable	Categories (No)		3-year OS	*p*-value *(HR)*	3-year LRC	*p*-value *(HR)*	3-year MFS	*p*-value *(HR)*
Age	< 65	159	21.3%	**0.02**	57.5%	0.40	36.3%	0.23
	≥65	178	16.1%	***(1.34)***	54.0%	*(1.21)*	40.3%	*(0.81)*
Sex	M	260	16.1%	**0.02**	55.6%	0.64	36.7%	0.23
	F	77	29.6%	***(0.70)***	57.5%	*(0.88)*	44.9%	*(0.77)*
Histology	squamous	206	14.8%	0.02 *	49.1%	0.02 *	41.2%	0.23 *
	adeno	50	31.9%	***(0.64)***	82.4%	***(0.36)***	35.5%	*(1.32)*
	NOS	81	24.6%		52.3%		32.8%	
T stage	1–2	120	22.2%	**< 0.001**	62.4%	0.04	42.9%	**0.02**
	3–4	201	16.6%	***(1.61)***	48.3%	*(1.61)*	36.8%	***(1.57)***
	N/A	16						
N stage	0–1	71	17.6%	0.86	64.2%	0.13	36.9%	0.36
	2–3	250	20.2%	*(1.03)*	54.2%	*(1.59)*	41.4%	*(1.23)*
	N/A	16						
M stage	0	260	21.2%	**< 0.001**	55.2%	0.95	47.9%	***< 0.001***
	1	68	8.6%	***(1.78)***	65.7%	*(1.02)*	0.0%	***(8.7)***
	N/A	9						
ZUBROD	0–1	306	19.6%	**0.015**	55.6%	0.23	37.4%	0.43
	2–3	31	19.4%	***(1.72)***	61.3%	*(1.60)*	60.9%	*(0.72)*
Radiotherapy	< 60 Gy	189	10.1%	**< 0.001**	49.6%	**0.004**	19.4%	**< 0.001**
	≥60 Gy	148	30.6%	***(0.38)***	61.5%	***(0.52)***	35.9%	***(0.29)***
Smoking status	never	22	61.6%	**< 0.001**	81.5%	**0.04**	46.8%	0.21
	ever	314	17.3%	***(3.66)***	53.2%	***(4.45)***	38.2%	*(1.54)*
Chemotherapy	no	124	12.7%	**< 0.001**	53.5%	**0.02**	42.0%	**0.009**
	yes	213	23.7%	***(0.43)***	57.1%	***(0.55)***	28.8%	***(0.61)***

*comparison between squamous cell cancer and adenocarcinoma

Significance (*p*-value, HR) refers to the univariable Cox regression analysis, 3-year OS, LRC and MFS in the respective groups are also presented

The median age of the patients was 65 years, there were 260 males (77.2%) and 77 females (22.8%). Squamous cell cancer was diagnosed in 206 patients (61.1%), adenocarcinoma in 50 patients (14.8%) and NOS in 81 individuals (24.1%). Most of the patients (90.8%) had a good performance status (ZUBROD 0–1). An advanced primary tumor stage (T3–4) was diagnosed in 201 patients (59.6%), while 250 patients (74.2%) had an advanced (N2–3) nodal stage. In 68 individuals (20.2) distant metastases were present at the diagnosis (M1).

The proportion of the patients according to the 8th edition of AJCC staging system was as follows: stage I, 16 patients (4.7%); stage II, 16 patients (4.7%); stage IIIA, 106 patients (31.5%); stage IIIB, 123 patients (36.6%); stage IV, 71 patients (21%); and uncertain stage, 5 patients (1.5%). We noted that all patients in stages I and II who were included in the present study were excluded from surgery due to comorbidities or lack of consent: they were treated with radiotherapy alone (stereotactic treatment, 60 Gy in 3–5 fractions). The majority of the patients included in the study (*N* = 229, 68.1%) were in clinical stages IIIA and IIIB. Nevertheless, this group was heterogeneous with respect to general performance and extent of disease and depending on these features 133/229 patients in this group (58.0%) received radiotherapy with curative intent, compared to 96/229 patients (42.0%) who were administered palliative thoracic radiotherapy. We note that several patients in this stage were treated with surgery and, thus, excluded from the present study. Therefore, the group is not representative of general cohorts of the patients with stage IIIA-IIIB disease.

Only 4 out of 71 patients with stage IV disease were administered radiotherapy with curative intent: this treatment was restricted to individuals in good general performance with oligometastatic disease, all others had palliative thoracic radiotherapy.

### Radiation treatment

In general, 3 D radiation treatment planning was used, except for individuals with an impaired performance status (ZUBROD 2–3; Table 1) treated with palliative intent, where 2 D planning was allowed. PET/CT was used for 3D radiation treatment planning in all 148 individuals treated with curative intent. For patients treated with palliative intent PET/CT was used for staging and/or planning whenever considered relevant for proper assessment of the extent of disease. Patients were immobilized in a vacuum mattress or thermoplastic mask and treated in the supine position on a linear accelerator.

Thoracic radiotherapy with curative intent (range, 60–74 Gy; median, 69.2 Gy; mean 68.2 Gy) was given to 148 patients (43.9%). The median fraction dose in this group was 1.8 Gy, the mean fraction dose was 2.4 Gy, and the range of the fraction dose was 1.8–20 Gy. The gross tumor volume (GTV) in curatively treated patients included primary tumor and involved mediastinal nodes. CTV added a margin of 0.5–1 cm for subclinical disease, while ITV accounted for respiratory motion and setup uncertainties. Elective nodal radiotherapy was not used in this group.

Thoracic radiotherapy with palliative intent (range 20–54 Gy, median 20 Gy, mean 24.6 Gy) was administered to 189 patients (56.1%). The median fraction dose in this group was 4.0 Gy, and the mean fraction dose was 3.7 Gy. The GTV in palliatively treated patients encompassed the primary tumor and massively enlarged nodes that contributed to symptoms of disease. We note that radiation treatment techniques and dose-fractionation did not change in Gliwice over the period of the present study.

### Chemotherapy

Chemotherapy was prescribed to 213 patients.

Of 148 patients treated with curative intent 131 (88.5%) received induction chemotherapy, i.e., 2–4 courses of a platinum- based doublet (median 4, range 1–6) were prescribed before the administration of radiotherapy. Such a treatment schedule, was demanded by the protocol of the institutional research project on dose- fractionation in sequential treatment.

Of 189 patients treated with palliative intent 82 (43.4%) received chemotherapy as a part of the first-line treatment (median, 4 cycles; range, 1–4). A platinume- based doublet was the most commonly prescribed palliative regimen. In addition, 36 patients (19.0%) treated with palliative intent received chemotherapy at disease progression.

Overall, cis-platine 80 mg/m2 on day 1 and vinorelbine 30 mg/m2 day 1, 8 doses given every 21 days was the most frequently prescribed regimen (155/213 patients, i.e., 72.8%). Cis-platine and gemcitabine were prescribed in 14 patients (6.5%), Cis-platine and etoposide were also prescribed in 14 patients (6.5%). A carboplatine- based doublet was prescribed in 18 patients (8.4%) and other chemotherapy regimens (mostly navelbine in monotherapy) in the remaining 12 individuals (5.6%). In general, chemotherapy was prescribed sequentially with chemotherapy with only 2 individuals (0.9%) receiving concurrent treatment.

### Blood markers

In general, the selection of biomarker testes was based on a literature search with a particular focus on markers of hypoxia, angiogenesis, immune responses and proliferation, i.e. the processes that determine the tumor response to radiotherapy and, hence, the long-term outcome of radiotherapy-based treatment. Of several markers that were suggested as prognostic biomarkers in lung cancer, we selected the following: OPN, VEGF, EPO, HMGB1, IGF1–1, PDGF.

Osteopontin (OPN) is a protein that interacts with multiple cell surface receptors involved in several physiological and pathological processes including tumorigenesis, immune responses, inflammation, wound healing, bone turnover, and ischemia [11]. It was demonstrated that OPN might be a useful biomarker in lung cancer therapy [12]. Its prognostic significance was demonstrated not only in lung cancer patients treated with radiotherapy [13] but also in patients with early stage disease treated with surgery [14] or in advanced cases treated with chemotherapy [15].

Vascular endothelial growth factor (VEGF) is a protein involved in the formation of blood vessels. As a biomarker related to angiogenesis, it has apparent prognostic potential in cancer therapy [16]. The prognostic utility of VEGF was shown in patients with lung cancer treated with surgery [17], radiotherapy [18] and chemotherapy [19].

Erythropoetin (EPO) is a protein that is secreted by the kidney in response to cellular hypoxia, it stimulates erythropoiesis. The involvement of this biomarker in response to hypoxia is particularly interesting considering the importance of hypoxia for radiation treatment. Additionally, EPO may have a direct growth-promoting effect on cancer cells, a phenomenon of clinical importance in radiotherapy [20]. The prognostic utility of erythropoetin in therapy for lung cancer was demonstrated in patients treated with radiotherapy [21] and surgery [22].

High mobility group box 1 protein (HMGB1) regulates transcription in cells and mediates immune reactions, which are processes of major importance in tumor progression [23]. This marker was tested mainly for the purpose of the early diagnosis of lung cancer [24].

Insulin-like growth factor 1 (IGF-1) is a mediator of the effects of growth hormone involved in several growth-promoting effects, including the proliferation of cancer cells [25]. Its prognostic utility in therapy for lung cancer was suggested by some authors [26–28], although the results are somewhat conflicting.

Platelet-derived growth factor (PDGF) regulates cell growth and division and plays a significant role in angiogenesis) [29]. Its prognostic utility in therapy for lung cancer has been investigated by a few authors [30, 31].

The concentrations of the selected proteins in serum (plasma in the case of OPN) were measured by enzyme-linked immunosorbent assay (ELISA) using a Wallac Victor Multi-label Counter. For the assessment of the EPO concentration, chemiluminescence was used. Table 2 briefly summarizes the methodology used to assess the biomarker concentration. Human serum/plasma was prepared by centrifuging whole blood at 1000 *g* for 15 min.

**Table 2:** Description of the methodology used to assess the biomarker concentration

Marker	Test name	Company	Method	Material /storage	Measurement units
OPN	Quantikine ELISA Human Osteopontin (OPN)	R&D Systems	ELISA	plasma/−80 °C/	ng/ml
VEGF	RayBio Human VEGF ELISA Kit	RayBiotech,Inc.	ELISA	serum/−80 °C	pg/ml
EPO	Immulite 2000 EPO	Siemens	chemiluminescence	serum/−80 °C	mIU/ml
HMGB1	HMGB1 ELISA	IBL International GMBH	ELISA	serum/−80 °C	ng/ml
IGF1	IGF-I ELISA	DRGInstruments GmbH	ELISA	serum/−80 °C	ng/ml
PDGF	RayBio Human PDGF-BB ELISA Kit	RayBiotech,Inc.	ELISA	serum/−80 °C	pg/ml

The blood samples were drawn before treatment, i.e., at the onset of induction chemotherapy, or before the first fraction of radiotherapy in the case of radiation treatment alone. The serum was stored in a refrigerator at − 80 °C.

Blood samples were prospectively collected and all enrolled patients gave their informed consent to participate. Considering limited funds for this research an interim analysis of the prognostic utility of the selected biomarkers was performed after enrolment of the initial 80–100 patients and further measurements were abandoned, providing a lack of independent prognostic potential in the pilot series. For this reason, IGF 1–1 measurements were abandoned considering the strong correlation of this marker with the TNM stage. Likewise, PDGF measurements were abandoned considering the strong correlation of this marker with the T stage and tumor pathology. Some measurements of OPN, VEGF, EPO and HMGB1 are missing (Table 3), mostly due to temporary limitations in the supply of the reagents.

**Table 3:** Influence of the selected biomarkers on overall survival (OS), locoregional control (LRC) and metastases-free survival (MFS)

Biomarker *(units)*	Cut-off	(No)	3-year OS	*p*-value *(HR)*	3-year LRC	*p*-value *(HR)*	3-year MFS	*p*-value *(HR)*
OPN (*n* = 290) *(ng/ml)*	< 104	145	28.0%	**< 0.001**	60.5%	0.13	41.7%	0.10
	≥104	145	16.6%	***(1.74)***	55.4%	*(1.45)*	40.2%	*(1.36)*
VEGF (*n* = 246) *(pg/ml)*	< 362	122	31.6%	**0.001**	57.8%	0.66	42.7%	0.20
	≥362	122	15.9%	***(1.67)***	60.1%	*(1.12)*	36.1%	*(1.30)*
EPO (*n* = 223) *(mIU/ml)*	< 16.1	107	26.0%	**0.04**	62.6%	0.47	48.2%	**0.02**
	≥16.1	116	15.6%	***(1.38)***	62.1%	*(1.24)*	26.3%	***(1.67)***
HMGB1 (*n* = 206) *(ng/ml*)	< 2.85	70	26.7%	0.64	68.9%	0.39	38.9%	0.64
	≥2.85	206	24.6%	*(1.09)*	54.9%	*(1.30)*	44.2%	*(1.11)*
IGF 1–1 (*n* = 98) *(ng/ml)*	< 99	49	16.3%	0.87	55.1%	0.31	37.6%	0.57
	≥99	49	9.2%	(0.97)	21.9%	*(1.44)*	33.7%	*(1.19)*
PDGF (*n* = 85) *(pg/ml)*	< 249	42	20.2%	0.29	26.5%	**0.013**	37.1%	0.19
	≥249	43	13.9%	*(1.28)*	73.0%	***(0.31)***	34.5%	*(1.57)*

Significance (*p*-value, HR) refers to the univariable Cox regression analysis, 3-year OS, LRC and MFS in the respective groups are also presented

We note that all of the markers selected have previously been studied as prognosticators [5–31], but only some of these studies recruited the patients treated with radiotherapy [8–10, 13, 15, 18, 21, 32]. Furthermore, in several studies only one or two biomarkers were studied, thus the independent prognostic value of these biomarkers (confronted with the other markers) could not be accurately evaluated. Likewise, not all of the studies incorporated clinical prognosticators among the variables studies. For this reason the present analysis accounts for both factors (i.e., the potential clinical prognosticators and the potential blood biomarkers).

### Statistical analysis

Overall survival (OS), locoregional control (LRC) and metastasis-free survival (MFS) were the major endpoints of the study (Table 1). The same methodology was used in the analysis of these endpoints. The patients were categorized according to the concentrations of the markers, using the median concentration as a cut-off. Such selection was driven by the practical utility and simplicity of this approach in which the same cut-off was used for different endpoints (OS, LRC, MFS). Several other variables of possible prognostic value were also considered in the analysis including age, gender, pathology, TNM stage, ZUBROD performance status, smoking status, use of chemotherapy and use of curative vs. palliative doses of radiotherapy.

Survival curves (OS, LRC, MFS) and the corresponding 3-year actuarial survival rates were estimated using the Kaplan-Meier method. Survival curves were compared using log-rank test, and a *p*-value of < 0.05 was considered significant. A Cox proportional hazard regression model was used to estimate hazard ratios (HR). The cut-off points for biomarker concentration were selected as the median value for a given marker.

The variables that appeared to significantly influence the endpoints of the study (OS, LRC and MFS) were selected for a multivariable analysis. Multivariable analysis was performed using the Cox proportional hazard regression model, and the backward selection method was used to select the important variables to be included in the model. In general, missing data were deleted in a univariate analysis and substituted by means in a multivariable study. Several variants of the basic analysis were considered, including subset analysis, considering of the continuous variables instead of dichotomizing them, and substitution of the missing data by means vs. deleting them: the most important outcomes of these variants (particularly those that may influence the qualitative outcome of this research) are presented in the text. Because locoregional recurrence and development of distant metastases are considered competitive reasons for treatment failures only the analysis of OS was performed in the subgroups; likewise, the variants of LRC and MFS analysis are not discussed.

## Results

### The outcome in a heterogeneous group of 337 patients

The 3-year actuarial overall survival in a whole group of 337 patients was 19.6% (10.1% for palliative treatment and 30.6% for curative treatment), Table 1. Of 16 variables studied age < 65 years (vs. ≥65 years), female sex (vs. male sex), adenocarcinoma histology (vs. squamous), tumor stage 1–2 (vs. stages T3–4), M0 stage (vs. M+ stage), performance status ZUBROD 0–1 (vs. 2–3), use of curative doses of radiotherapy (vs. doses< 60 Gy), never smoking status (vs. smoking), and use of chemotherapy (vs. no chemotherapy) were significantly associated with favorable overall survival (Table 1). Of the 6 biochemical markers studied OPN concentration below the median of 104, VEGF concentration below the median of 362 and EPO concentration below the median of 16.1 were also significantly associated with favorable overall survival (Table 3). Table 1 and Table 3 present the results of the univariable Cox regression analysis of OS, LRC and MFS and 3-year actuarial OS, LRC and MFS estimates in each of the categories analyzed. The tables also illustrate how the respective variables were dichotomized.

**Fig. 1:**
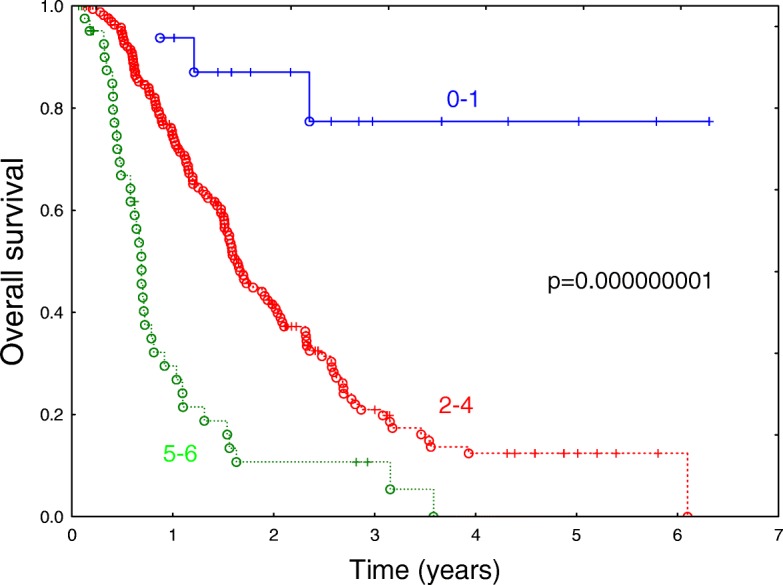
Overall survival in the heterogeneous group of patients with non-small cell lung cancer, depending on number of 6 independent risk features (cigarette smoking, lack of chemotherapy, palliative doses of radiotherapy, high OPN concentration, advanced T stage and high VEGF concentration). Missing data were deleted (*n* = 227). The differences between the patients with 0–1, 2–4 and 5–6 risk features are highly significant (*p* < 0.00001)

The multivariable analysis (Table 4) revealed that out of several variables studied, six features: namely,cigarette smoking, lack of chemotherapy, palliative doses of radiotherapy, high OPN concentration, advanced T stage and high VEGF concentration, had a highly significant and independent negative influence on overall survival in the group of 337 patients. Figure 1 illustrates the overall survival in a group of patients with non-small cell lung cancer depending on number of the defined risk features (cigarette smoking, lack of chemotherapy, palliative doses of radiotherapy, high OPN concentration, advanced T stage and high VEGF concentration). The differences between the patients with 0–1, 2–4 and 5–6 risk features were highly significant (*p* < 0.00001). Deletion of the missing data vs. replacing them with means did not change the qualitative outcome of this part of the analysis.

**Table 4:** Results of the multivariable study of the influence of selected variables on overall survival in 337 patients with non-small cell lung cancer. Backward selection was used to select the variables

Variable	HR (95% CI)	*p*-value
Ever smoking vs. never smoking	3.62 (1.69–7.76)	0.000955
No chemo vs. chemo	2.03 (1.53–2.70)	0.000001
Palliative RT vs. curative RT	2.00 (1.49–2.68)	0.000004
OPN ≥104 ng/ml vs. OPN < 104 ng/ml	1.67 (1.27–2.20)	0.000285
T 3–4 vs. T1–2	1.52 (1.15–2.00)	0.003362
VEGF1 ≥ 362 pg/ml vs. VEGF< 362 pg/ml	1.51 (1.11–2.05)	0.007952

Consideration of continuous variables, instead of dichotomous variables, revealed, however, that in a multivariable analysis of overall survival, the VEGF concentration lost an independent significant prognostic influence on overall survival in favor of patient age. The variables remaining significant in this model were: OPN concentration (*p* < 0.00001), intent of radiotherapy (*p* = 0.00002), smoking status (*p* = 0.0007), use of chemotherapy (*p* = 0.0001), T stage (*p* = 0.003) and patient age (*p* = 0.02). Such an outcome can be explained by the correlation of VEGF and smoking (higher VEGF in smokers) and T stage (increase of VEGF concentration in advanced cases). Additionally, VEGF concentrations tended to be log-normally distributed. When logarithmic transformation of the VEGF concentration was applied, the significance of logarithmically transformed VEGF measurement increased, approaching the threshold for significance in a multivariable model.

Four variables (adenocarcinoma, use of curative doses of radiotherapy, never smoking status, use of chemotherapy) were significantly associated with favorable LRC. A low PDGF concentration (< 249 pg/ml) was also associated with favorable LRC, but this was largely due to the correlation of PDGF concentration with other prognosticators (tumor pathology, T stage). The multivariable analysis of dichotomized variables revealed that only the use of curative radiotherapy and smoking status were significantly and independently associated with LRC.

Of five variables that significantly influenced MFS in the univariable study (T stage, M stage at diagnosis, use of curative radiotherapy use of chemotherapy, and EPO concentration) only the use of palliative radiotherapy significantly and independently influenced MFS (*p* < 0.00001, HR = 3.45), with patients receiving palliative treatment at higher risk for developing distant metastases.

### The outcome in a subset of patients with curative radio-chemotherapy or radiotherapy

The patients who were treated with curative radio-chemotherapy or radiotherapy (*N* = 148) created an important, distinct, and relatively homogeneous subset, among the patients studied. Most of the patients in this group were administered induction chemotherapy (131/148, 88.5%), and 17 individuals (11.5%) had radiotherapy alone. The 3-year actuarial overall survival in this group was 30.6% and was significantly influenced by the following variables: gender, pathology, N-stage, smoking status, VEGF concentration, and EPO concentration (Table 5). The 3-year actuarial overall survival was particularly favorable in never smoking patients (64.2%) and in patients with adenocarcinoma (51.0%). The multivariable analysis revealed a significant and independent influence on survival of the following dichotomized variables:

1. pathology (squamous vs. nonsquamous, *p* = 0.003, HR 0.52, 95% CI 0.33–0.80)
2. EPO concentration (< 16.1 vs ≥16.1, *p* = 0.001, HR = 2.33, 95% CI 1.37–3.95)
3. VEGF concentration (< 362 vs. ≥362, *p* = 0.007, HR = 1.87, 95% CI 1.19–2.96)

**Table 5:** Variables that had significant influence on overall survival in a subset of 148 patients with curative radio-chemotherapy or radiotherapy (univariate analysis)

Variable	Categories (No)		3-year OS	*p*-value *(HR)*
Gender	M	111	25.6%	**0.02**
	F	37	45.0%	***(0.58)***
Pathology	squamous	90	22.0%	0.005 *
	adeno	22	51.0%	***(0.42)***
	NOS	36	39.9%	
N stage	0–1	40	18.8%	**0.02**
	2–3	107	34.4%	***(0.60)***
	N/A	1		
Smoking status	never	12	64.2%	**< 0.04**
	ever	136	28.2%	***(2.76)***
VEGF (*n* = 125)	< 362 pg/ml	62	38.7%	**0.006**
	≥362 pg/ml	63	22.7%	***(1.89)***
EPO (*n* = 96)	< 16.1 mlU/ml	51	40.3%	**0.008**
	≥16.1 mlU/ml	45	15.9%	***(2.02)***

*comparison between squamous-cell cell cancer and adenocarcinoma

**Fig. 2:**
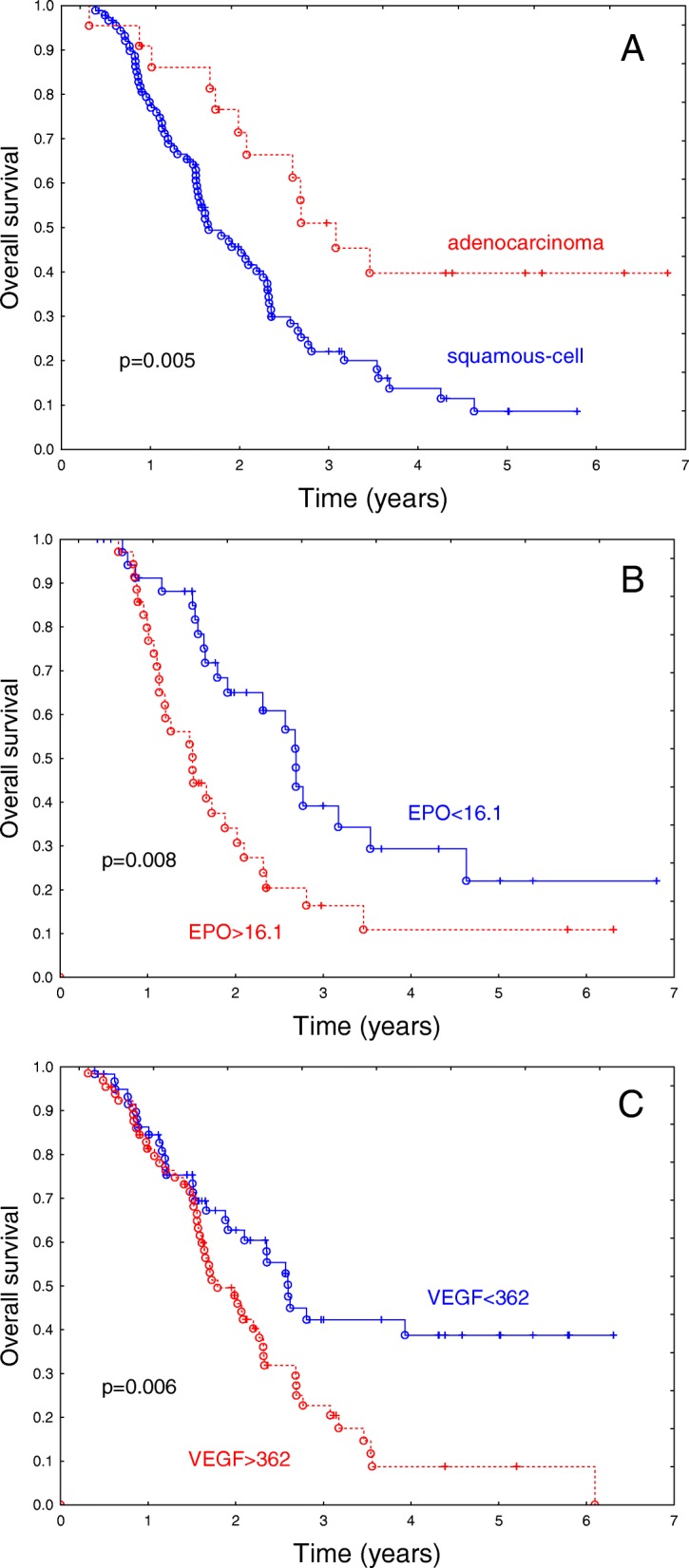
Influence of tumor pathology (**a**), EPO concentration (**b**) and VEGF concentration (**c**) on overall survival in a group of 148 patients with non-small cell lung cancer treated with curative radio-chemotherapy or radiotherapy. The differences in overall survival are highly significant (*p* = 0.005; *p* = 0.008; *p* = 0.006 in groups **a**, **b**, **c** respectively). Median EPO and VEGF concentration was selected as a cut-off

Figure 2 illustrates the influence of these variables on overall survival in the analyzed subset.

### The prognostic significance of blood markers vs. pathology of the tumor

Tumor pathology had a major influence on overall survival both in a whole group of 33 patients in the subset treated with curative intent (Table 1, Table 4). For this reason, the prognostic value of the markers tested was analyzed in subsets that were uniform with respect to tumor pathology.

**Fig. 3:**
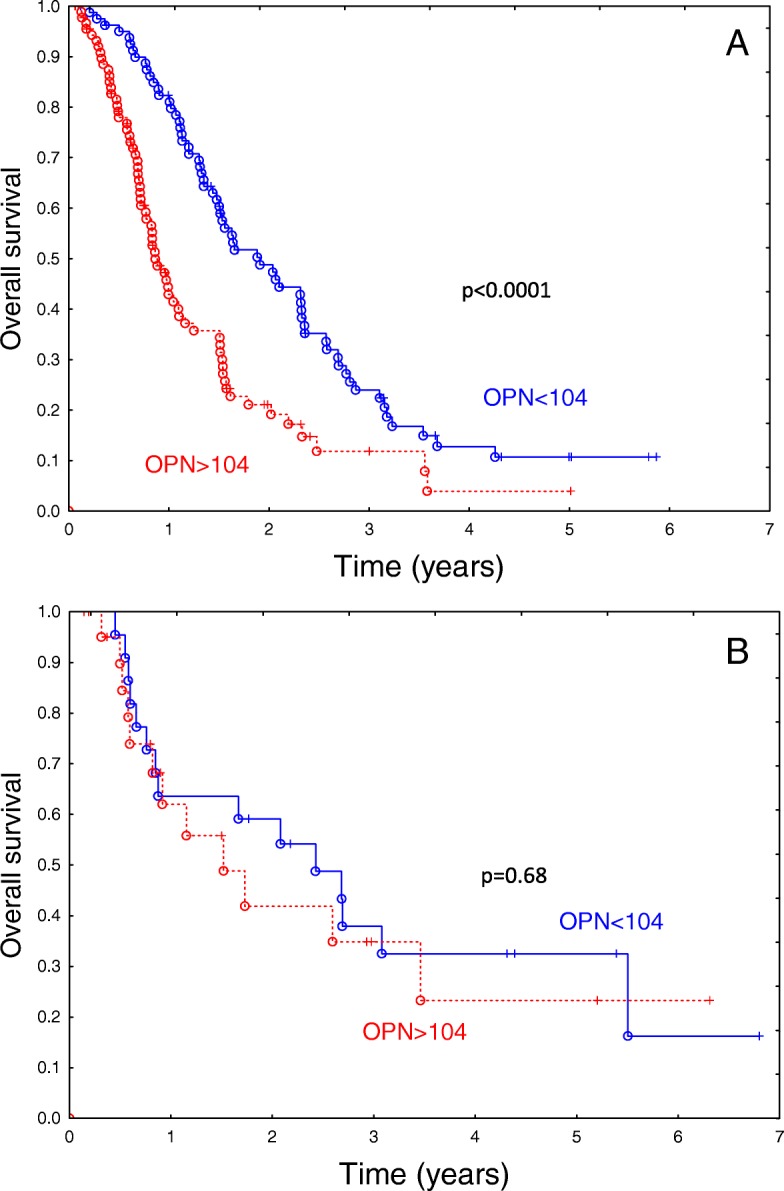
Influence of OPN concentration on overall survival in a group of 171 patients with squamous-cell cancer (**a**) and 44 patients with adenocarcinoma (**b**). The difference in overall survival according to biomarker concentration is highly significant among the patients with squamous cell cancer (*p* < 0.0001), but not among the patients with adenocarcinoma. 0.18

In the subset of patients with squamous cell cancer (*N* = 206) OPN had a highly significant impact on overall survival (*p* < 0.0001; HR 2.09; 95% CI, 1.468–2.99), (Fig. 3a). The EPO concentration also appeared to have a significant influence (*p* = 0.019; HR = 1.60; 95% CI, 18–2.36). However, an influence of VEGF and HMGB1 did not appear significant (*p* = 0.08, *p* = 0.90, respectively).

In contrast, in a subset of patients with nonsquamous histology (*N* = 131), only VEGF had a significant influence on survival (*p* = 0.02; HR = 1.78; 95% CI, 1.08–2.95), while OPN, EPO and HMGB1 did not appear to have a significant influence (*p* > 0.05). A similar qualitative outcome was found among the patients with adenocarcinoma (*N* = 44): VEGF tended to influence survival (*p* = 0.10), while the other markers did not appear to have a significant influence (Fig. 3b).

## Discussion

Despite of recent developments in therapy for advanced non-small cell lung cancer the overall survival in unselected groups of patients remains poor with a median of 1.3 years in the present series of 337 patients. The unfavorable prognosis does not, however, refer to all of the advanced cases. The median survival among the patients with curative radio-chemotherapy or radiotherapy was 2.1 years, and the 3-year survival in this group was 30.6%. This survival was further improved considering well-defined subsets of patients with curative treatment, particularly those with favorable prognostic features (Fig. 2).

Evaluation of the prognosis in routine clinical practice is, at present, based mostly on the stage of disease, tumor pathology and the ability to deliver curative doses of radiotherapy. The present analysis shows, however, that the use of blood serum proteins as biomarkers can considerably improve the prognostic ability of clinicians. When dichotomized, OPN and VEGF concentration and smoking status of the patients, (Fig. 1, Table 1, Table 3) appeared among the factors that strongly influence the prognosis. Interestingly, the prognostic utility of the blood serum markers as measured by the respective hazard ratios is comparable to the strongest clinical prognosticators known. It is not surprising that out of several markers studied OPN and VEGF appeared to be of greatest interest: both are related to tumor hypoxia, a factor that has major influence on outcome in radiotherapy. An independent prognostic influence of VEGF on survival seems, however, smaller than that of OPN. When analyzed as a continuous variable VEGF lost its independent prognostic value in favor of the other variables. This was, most likely, due to the correlation of VEGF with smoking status and T stage. Additionally, the distribution of VEGF measurements appeared log-normal, contributing to such outcomes. Clearly, the diverse outcomes of the basic analysis options show the limitations of the present study. Such diverse outcomes may, however, also indicate that blood biomarkers may, in some situations, become surrogates of clinical or pathological prognosticators that were unintentionally or deliberately not included in the model.

Most of the patients in the present series were current or former smokers; a small subset of the never smokers had, however, a very favorable prognosis (median survival not reached). While the molecular analysis of tumors was not systematically performed in the present series the data from the other series suggest a distinct characteristic of nonsmokers including the prevalence of tumors with EGFR mutations [32]. These features were linked to higher radiosensitivity of the tumors that might have been reflected by favorable survival in the present series of patients treated with radiotherapy. By contrast, smoking was related to a high frequency of KRAS mutations, which is a feature linked to impaired prognosis in patients treated with radiotherapy [33].

The subset of patients with curative radio-chemotherapy or radiotherapy is considerably more clinically homogeneous, than the unselected group. The present analysis suggests the prognostic utility of tumor pathology, EPO concentration and VEGF concentration that were dichotomized based on the median values. In particular, adenocarcinoma pathology favorably influenced survival (3-year survival of 51%; median survival of 3.1 years). The lack of independent prognostic significance of OPN in patients with curative radio-chemotherapy or radiotherapy was elucidated by the analysis of the prognostic significance of blood markers vs. pathology of the tumor; the utility of OPN as a prognostic marker appeared to be restricted to the patients with squamous histology. For patients with nonsquamous histology treated with curative radio-chemotherapy or radiotherapy VEGF appeared to be the most useful marker. This finding shows that the utility of given markers useful in a heterogeneous group of patients is not necessarily reproduced in more homogenous subsets.

The data presented conform reasonably well with large studies on the prognostic significance of plasma biomarkers in radiotherapy for non-small cell lung cancer [8–10]. Ostheimer et al. [8] analyzed a group of 55 patients treated with curative radiotherapy or radio-chemotherapy. They analyzed plasma concentrations of OPN, VEGF and CA IX (carbonic anhydrase IX). The study demonstrated that high pretreatment plasma levels of OPN, CA IX and VEGF were correlated with impaired prognosis in M0-stage NSCLC patients receiving radical radiotherapy, which is in a good concordance with the present results.

Dehing et al. [9] analyzed the survival of non-small-cell lung cancer patients treated with combined chemotherapy and radiation or radiotherapy alone. The analysis included the concentration of lactate dehydrogenase, C-reactive protein, osteopontin, carbonic anhydrase IX, interleukin (IL) 6, IL-8, carcinoembryonic antigen (CEA), and cytokeratin fragment 21–1. The statistical model was built on 106 patients and validated on 52 individuals. The performance of the prognostic model for survival that included clinico-pathological features improved markedly by adding two blood biomarkers: CEA and IL-6. We note that only 35% of the patients in this study had squamous-cell cancer. Therefore, we believe, that considering the outcome of the present study, the data presented by Dehing et al. [9] may not preclude the prognostic significance of osteopontin in patients with squamous-cell histology.

Recently Walker et al. [10] presented data on the discovery and validation of predictive biomarkers of survival for non-small cell lung cancer patients undergoing radical radiotherapy. Over 650 proteins were detected and quantified using a mass spectrometry discovery proteomics platform. Plasma samples from patients before and during radiotherapy who had survived over 18 months were compared to the same time points from patients who survived less than 14 months. Two of the proteins studied CRP (C-reactive protein) and LRG1 (leucine-rich-alpha-2 glycoprotein) gave a highly significant indication of extended survival. We note, however, that contrary to the studies by Ostheimer et al. [8], and Dehing et al. [9] and the present investigation, the analysis did not incorporate clinically relevant variables such as age, stage, and performance of the patients. Therefore, the proteins of interest (CRP and LRG1) should be confronted with other variables influencing survival in the future studies to evaluate their possible independent prognostic utility.

## Conclusions

Blood serum proteins appear to be clinically useful prognosticators of overall survival in radio-chemotherapy and radiotherapy for non-small cell lung cancer. In unselected groups, heterogeneous with respect to the intent of treatment and tumor histology the dichotomized concentration of OPN and VEGF emerged among the strongest independent prognostic factors next to smoking status, use of chemotherapy, doses of radiotherapy and tumor stage. In particular, the OPN concentration exhibited a prognostic power comparable to or higher than that of the strongest known clinical prognosticators.

When dichotomized, VEGF and EPO concentrations as well as tumor histology were found to be independent prognostic factors among the patients treated with curative doses of radiotherapy. The utility of OPN as a prognostic marker appeared to be restricted to patients with squamous histology. For patients with nonsquamous histology VEGF appeared to be the most useful marker.

## Abbreviations

CTV: Clinical target volume
EPO: Erythropoetin
GTV: Gross tumor volume
HMGB1: High mobility group box 1 protein
IGF-1: Insulin-like growth factor 1
LRC: Locoregional control
MFS: Metastases-free survival
OPN: Osteopontin
OS: Overall survival
PDGF: Platelet-derived growth factor
PET/CT: Positron emission tomography/computer tomography
VEGF: Vascular endothelial growth factor

## References

[1] Syrigos K, Fiste O, Charpidou A, Grapsa D (2018). Circulating tumor cell count as a predictor of survival in lung cancer. Crit Rev Oncol Hematol.

[2] Paci M, Maramotti S, Bellesia E, Formisano D, Albertazzi L, Ricchetti T, Ferrari G, Annessi V, Lasagni D, Carbonelli C (2009). Circulating plasma DNA as a diagnostic biomarker in non-small cell lung cancer. Lung Cancer.

[3] Suwinski R, Klusek A, Tyszkiewicz T (2012). Gene expression from bronchoscopy obtained tumour samples as a predictor of outcome in advanced inoperable lung cancer. PLoS One.

[4] Thunnisen E, van der Oord K, Bakker M (2014). Prognostic and predictive biomarkers in lung cancer: a review. Virchows Arch.

[5] Xu-Welliver M, Carbone DP (2017). Blood-based biomarkers in lung cancer: prognosis and treatment decisions. Transl Lung Cancer Res.

[6] Brothers JF, Hijazi K, Mascaux C, El-Zein RA, Spitz MR, Spira A (2013). Bridging the clinical gaps: genetic, epigenetic and transcriptomic biomarkers for the early detection of lung cancer in the post-National Lung Screening Trial era. BMC Med.

[7] Dai L, Tsay JC, Li J Yie TA, Munger JS, Pass H4 Rom WN, Zhang Y, Tan EM, Zhang JY. Autoantibodies against tumor associated antigens in the early detection of lung cancer. Lung Cancer 2016;99:172–179. 10.1016/j.lungcan.2016.07.018 27565936

[8] Ostheimer C, Bache M, Güttler A, Kotzsch M, Vordermark D (2014). A pilot study on potential plasma hypoxia markers in the radiotherapy of non-small cell lung cancer. Osteopontin, carbonic anhydrase IX and vascular endothelial growth factor. Strahlenther Onkol.

[9] Dehing-Oberije C, Aerts H, Yu S, De Ruysscher D, Menheere P, Hilvo M, van der Weide H, Rao B, Lambin P (2011). Development and validation of a prognostic model using blood biomarker information for prediction of survival of non-small-cell lung cancer patients treated with combined chemotherapy and radiation or radiotherapy alone (NCT00181519, NCT00573040, and NCT00572325). Int J Radiat Oncol Biol Phys.

[10] Walker MJ, Zhou C, Backen A (2015). Discovery and validation of predictive biomarkers of survival for non-small cell lung cancer patients undergoing radical radiotherapy: two proteins with predictive value. EBioMedicine..

[11] Wang KX, Denhardt DT (2008). Osteopontin: role in immune regulation and stress responses. Cytokine Growth Factor Rev.

[12] Zhang T1, Zhang DM, Zhao D, Hou XM, Liu XJ, Ling XL, Ma SC. The prognostic value of osteopontin expression in non-small cell lung cancer: a meta-analysis. J Mol Histol 2014;45(5):533–540. 10.1007/s10735-014-9574-3 24816798

[13] Ostheimer C, Bache M, Güttler A, Reese T, Vordermark D (2014). Prognostic information of serial plasma osteopontin measurement in radiotherapy of non-small-cell lung cancer. BMC Cancer.

[14] Boldrini L, Donati V, Dell'Omodarme M, Prati MC, Faviana P, Camacci T, Lucchi M, Mussi A, Santoro M, Basolo F, Fontanini G (2005). Prognostic significance of osteopontin expression in early-stage non-small-cell lung cancer. Br J Cancer.

[15] Isa S, Kawaguchi T, Teramukai S, Minato K, Ohsaki Y, Shibata K, Yonei T, Hayashibara K, Fukushima M, Kawahara M, Furuse K, Mack PC. Serum osteopontin levels are highly prognostic for survival in advanced non-small cell lung cancer: results from JMTO LC 0004. J Thorac Oncol. 2009;(9):1104–10. 10.1097/JTO.0b013e3181ae2844 19620934

[16] Patan S (2004). Vasculogenesis and angiogenesis. Cancer Treat Res Cancer Treatment and Research.

[17] Usuda K, Iwai S, Funasaki A, Sekimura A, Motono N, Ueda Y, Shimazaki M, Uramoto H (2018). Expression and prognostic impact of VEGF, CD31 and αSMA in resected primary lung cancers. Anticancer Res.

[18] Rades D, Setter C, Dunst J, Dahl O, Schild SE, Noack F (2010). Prognostic impact of VEGF and VEGF receptor 1 (FLT1) expression in patients irradiated for stage II/III non-small cell lung cancer (NSCLC). Strahlenther Onkol.

[19] Lissoni P, Rovelli F, Malugani F, Brivio F, Fumagalli L, Gardani GS (2003). Changes in circulating VEGF levels in relation to clinical response during chemotherapy for metastatic cancer. Int J Biol Markers.

[20] Sytkowski AJ. Does erythropoietin have a dark side? Epo signaling and cancer cells. Sci STKE. 2007;(395):pe38. 10.1126/stke.3952007pe38 17636183

[21] Rades D, Setter C, Dahl O, Schild SE, Noack F (2011). Prognostic impact of erythropoietin expression and erythropoietin receptor expression on locoregional control and survival of patients irradiated for stage II/III non-small-cell lung cancer. Int J Radiat Oncol Biol Phys.

[22] Saintigny P1, Besse B, Callard P, Vergnaud AC, Czernichow S, Colombat M, Girard P, Validire P, Breau JL, Bernaudin JF, Soria JC. Erythropoietin and erythropoietin receptor coexpression is associated with poor survival in stage I non-small cell lung cancer. Clin Cancer Res 2007;13(16):4825–4831. 10.1158/1078-0432.CCR-06-3061 17699861

[23] Sims GP, Rowe DC, Rietdijk ST, Herbst R, Coyle AJ (2010). HMGB1 and RAGE in inflammation and cancer. Annu Rev Immunol.

[24] Shang GH, Jia CQ, Tian H, Xiao W, Li Y, Wang AH, Dong L, Lin DJ (2009). Serum high mobility group box protein 1 as a clinical marker for non-small cell lung cancer. Respir Med.

[25] Singh P, Alex JM, Bast F (2014). Insulin receptor (IR) and insulin-like growth factor receptor 1 (IGF-1R) signaling systems: novel treatment strategies for cancer. Med Oncol.

[26] Guo C, Lu H, Gao W, Wang L, Lu K, Wu S, Pataer A, Huang M, El-Zein R, Lin T, Roth JA, Mehran R, Hofstetter W, Swisher SG, Wu X, Fang B (2013). Insulin-like growth factor binding protein-2 level is increased in blood of lung cancer patients and associated with poor survival. PLoS One.

[27] Shersher DD, Vercillo MS, Fhied C, Basu S, Rouhi O, Mahon B, Coon JS, Warren WH, Faber LP, Hong E, Bonomi P, Liptay MJ, Borgia JA (2011). Biomarkers of the insulin-like growth factor pathway predict progression and outcome in lung cancer. Ann Thorac Surg.

[28] Han JY, Choi BG, Choi JY, Lee SY, Ju SY (2006). The prognostic significance of pretreatment plasma levels of insulin-like growth factor (IGF)-1, IGF-2, and IGF binding protein-3 in patients with advanced non-small cell lung cancer. Lung Cancer.

[29] Farooqi AA, Siddik ZH (2015). Platelet-derived growth factor (PDGF) signalling in cancer: rapidly emerging signalling landscape. Cell Biochem Funct.

[30] Donnem T, Al-Saad S, Al-Shibli K, Andersen S, Busund LT, Bremnes RM (2008). Prognostic impact of platelet-derived growth factors in non-small cell lung cancer tumor and stromal cells. J Thorac Oncol.

[31] Shinohara ET, Gonzalez A, Massion PP, Olson SJ, Albert JM, Shyr Y, Carbone DP, Johnson DH, Hallahan DE, Lu B. PDGFR-beta expression in small cell lung cancer patients. Int J Radiat Oncol Biol Phys 2007; 1;67(2):431–437. 10.1016/j.ijrobp.2006.08.060 17236966

[32] Yagishita S, Horinouchi H, Taniyama T, Nakamichi S, Kitazono S, Mizugaki H, Kanda S, Fujiwara Y, Nokihara H (2015). Epidermal growth factor receptor mutation is associated with longer local control after definitive chemoradiotherapy in patients with stage III nonsquamous non-small-cell lung cancer. Int J Radiat Oncol Biol Phys.

[33] Mak RH, Hermann G, Lewis JH, Aerts HJ, Baldini EH, Chen AB, Colson YL, Hacker FH, Kozono D (2015). Wee JO et al outcomes by tumor histology and KRAS mutation status after lung stereotactic body radiation therapy for early-stage non-small-cell lung cancer. Clin Lung Cancer.

